# Cognition, behaviour and academic skills after cognitive rehabilitation in Ugandan children surviving severe malaria: a randomised trial

**DOI:** 10.1186/1471-2377-11-96

**Published:** 2011-08-04

**Authors:** Paul Bangirana, Peter Allebeck, Michael J Boivin, Chandy C John, Connie Page, Anna Ehnvall, Seggane Musisi

**Affiliations:** 1 Department of Psychiatry, Makerere University College of Health Sciences, Kampala, Uganda; 2 Department of Public Health Sciences, Karolinska Institutet, Stockholm, Sweden; 3 International Neurologic and Psychiatric Epidemiology Program, Michigan State University, East Lansing, MI, USA; 4 Neuropsychology Section, Department of Psychiatry, University of Michigan, Ann Arbor, MI, USA; 5 Department of Pediatrics, University of Minnesota, Minneapolis, MN, USA; 6 Department of Statistics and Probability, Michigan State University, East Lansing, MI, USA; 7 Institute of Clinical Neurosciences, Department of Psychiatry, Gothenburg University, Gothenburg, Sweden; 8 Psychiatric Clinic of Varberg, Halland County Council, Sweden

## Abstract

**Background:**

Infection with severe malaria in African children is associated with not only a high mortality but also a high risk of cognitive deficits. There is evidence that interventions done a few years after the illness are effective but nothing is known about those done immediately after the illness. We designed a study in which children who had suffered from severe malaria three months earlier were enrolled into a cognitive intervention program and assessed for the immediate benefit in cognitive, academic and behavioral outcomes.

**Methods:**

This parallel group randomised study was carried out in Kampala City, Uganda between February 2008 and October 2010. Sixty-one Ugandan children aged 5 to 12 years with severe malaria were assessed for cognition (using the Kaufman Assessment Battery for Children, second edition and the Test of Variables of Attention), academic skills (Wide Range Achievement Test, third edition) and psychopathologic behaviour (Child Behaviour Checklist) three months after an episode of severe malaria. Twenty-eight were randomised to sixteen sessions of computerised cognitive rehabilitation training lasting eight weeks and 33 to a non-treatment group. Post-intervention assessments were done a month after conclusion of the intervention. Analysis of covariance was used to detect any differences between the two groups after post-intervention assessment, adjusting for age, sex, weight for age z score, quality of the home environment, time between admission and post-intervention testing and pre-intervention score. The primary outcome was improvement in attention scores for the intervention group. This trial is registered with Current Controlled Trials, number ISRCTN53183087.

**Results:**

Significant intervention effects were observed in the intervention group for learning mean score (SE), [93.89 (4.00) vs 106.38 (4.32), *P *= 0.04] but for working memory the intervention group performed poorly [27.42 (0.66) vs 25.34 (0.73), *P *= 0.04]. No effect was observed in the other cognitive outcomes or in any of the academic or behavioural measures.

**Conclusions:**

In this pilot study, our computerised cognitive training program three months after severe malaria had an immediate effect on cognitive outcomes but did not affect academic skills or behaviour. Larger trials with follow-up after a few years are needed to investigate whether the observed benefits are sustained.

**Trial registration:**

ISRCTN: ISRCTN53183087

## Background

Cognitive deficits are common in children who have suffered from severe malaria having neurological involvement [1]. Severe malaria with neurological involvement (MNI) manifests as; deep coma (i.e. cerebral malaria), seizures, impaired consciousness, prostration and agitated behaviour [2,3]. Commonly affected cognitive functions are memory, attention, visual spatial ability, learning and language [4-10]. With cognitive impairment rates of between 14 to 26%, it is estimated that over 200,000 children in sub-Saharan Africa are left with impaired cognition annually after an episode of MNI [6,9]. Moreover, the occurrence of these cognitive impairments and their severity appears to be more evident two years later [9] suggesting that cognitive impairment becomes more apparent after a few years as children are required to perform more complex cognitive tasks. Psychopathologic behaviour are also increasingly being recognised in children who have suffered from MNI with hyperactive behaviours, conduct problems, autism like behaviours and other behavioural problems being reported in recent studies [11,12].

Malaria is thus a leading cause of neuro-disability in sub-Saharan Africa [1] and better interventions for child survivors are necessary [13]. Some of the suggested interventions include computerised cognitive rehabilitation training, caregiver training, early child education, nutritional support, physical therapy and speech therapy [13]. Computerised cognitive rehabilitation training (CCRT) may offer the best solution as it can be programmed to target specific functions, has proven effective in different forms of brain injury in both children and adults [14-20] and brings about physiological changes in the brain like increased brain activity, dopamine availability and changes in white matter structure [21-24]. Increased brain activity, availability of dopamine in the prefrontal areas and white matter integrity are in turn associated with performance of cognitive functions like working memory and executive functions [25-28].

In one of the pioneering applications of CCRT in sub-Saharan, children who suffered from cerebral malaria approximately four years earlier were randomised to either CCRT or a non-treatment group [29]. Post-intervention assessments showed improved memory, learning, psychomotor speed and internalising behaviour in the intervention group. This study however had some limitations. Firstly it was carried out almost four years after the illness yet studies indicate that cognitive test performance is poorer two years after the illness compared to controls [9]. This may probably explain why there was no improvement in visual attention yet impaired attention was the only cognitive deficit observed in these children two years earlier [9]. It is thus possible that interventions done some years after the illness may have little benefit since the cognitive impact of the disease is more apparent much later. Early interventions may stall the development of these deficits. Secondly, a computerised test of cognition was used and given that the intervention was also computerised, the observed benefits may have been a consequence of the familiarity with the computer.

We present a study addressing the above limitations whose objective was to evaluate the effect of CCRT given immediately after recovery from severe malaria (at three months) on cognition, behaviour and academic skills. Inclusion of academic skills will provide more information to an ongoing debate whether CCRT improves performance in other functional skills that are not directly trained by the intervention [30,31].

## Methods

### Study population and recruitment

Children were recruited from Mulago Hospital, the National Referral Hospital of Uganda, and from Nsambya, Rubaga and Mengo Hospitals all located in Kampala, Uganda's Capital City. Recruitment and follow up was done between February 2008 and October 2010. The latter three are large private mission hospitals. Participants were children aged 5 to 12 years presenting with a fever and *Plasmodium falciparum *on blood smears and one or more of the following; 1) a seizure lasting over 15 minutes or repeated seizures, 2) impaired consciousness (Glasgow coma scale score of 14 and below), 3) unarousable coma with normal cerebrospinal fluid findings. Children meeting these three criteria were categorised as malaria with seizures, malaria with impaired consciousness or cerebral malaria respectively. A physical examination and medical history were done on admission. The exclusion criteria were: history of or present meningitis or encephalitis, prior central nervous system infection, sickle cell disease, epilepsy, history of developmental delay and history of hospitalization for malnutrition.

**Figure 1:**
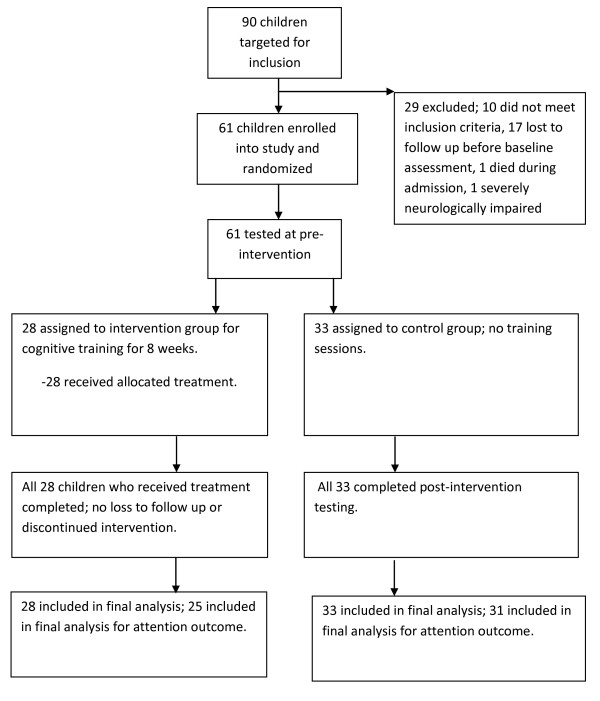
**Flow of participants in the study**.

Ninety children were recruited for the study. Of these, 10 did not meet the above inclusion criteria and were excluded and one died during admission leaving 79 children for follow-up. At discharge, home directions and telephone contacts were obtained from the parents/caregivers and appointment dates given for the baseline assessment three months later. In the interim period, a home visit was made to assess the quality of the home environment. By the three months hospital visit, 18 children had either withdrawn from the study or had been lost to follow-up or were neurologically impaired to participate in the study leaving 61 children who were given assessments of cognition, behaviour and academic skills. At the completion of these assessments, stratified randomisation for the three MNI groups (cerebral malaria, malaria with seizures and malaria with impaired consciousness) to either the CCRT group (N = 28) or the non-treatment group (N = 33) was done (see figure 1). A table of random numbers was used for group allocation. Post-intervention assessment was done a month after the intervention training. Based on our earlier study where the mean difference in attention between the two groups was 0.18 with a standard deviation of 0.24 [9], a study with a power of 80% would require 30 children in each arm. The above sample size gives a power of 78%.

Written informed consent was obtained from the parents or guardians of study participants and assent from children aged seven years and older. Ethical approval for this study was granted by the Institutional Review Board for Human Studies at Makerere University College of Health Sciences and the Uganda National Council for Science and Technology.

### Intervention

The CCRT package used was Captain's Log software [32] consisting of 35 multi-level brain-training exercises designed to help develop and remediate a wide range of cognitive skills. Fifteen of the 35 possible brain-training exercises were chosen for this study. The criteria for deciding which exercises to include were: (1) having little or no verbal instructions so that children with poor grasp of English would benefit and (2) having simple or few movements with the track-ball. Pretesting demonstrated that Ugandan children who were, for the most part unfamiliar with computers, would be more comfortable using a track-ball than a mouse, particularly if required movements were not large.

Four exercises were chosen to train attention; (1) scanning reaction/inhibition (clicking the mouse if the color of several varying images matches the color of the screen's border), (2) stimulus reaction time (the player is required to click the mouse once if the "target" image appears), (3) stimulus reaction/fields (the player is required to move the mouse and click it over the "target" image), and (4) stimulus reaction/inhibition (clicking the mouse if the color of several random images appearing one at a time matches the color of the screen's border).

The memory training exercises were; (5) conceptual (finding the missing part of a sequence from several choices), (6) logical sequences (finding and clicking on targets in the correct sequence), (7) size discrimination (clicking on target objects in order according to size), and (8) symbolic display match (selecting and placing targets in the correct box based on various rules).

Visuomotor Skills was trained by three exercises; (9) visual categorization (clicking on object that appears from behind a door according to the category rule); (10) visual response time (watching a grid of targets and clicking on any that changes), (11) visuospatial memory (searching for and find matching objects in a grid).

The four exercises for reasoning were; (12) concept logic (figuring out the secret rule in a number of images), (13) match logic (deciding whether images match or not), (14) picture logic (clicking on the target among foils), and (15) sequential logic (understanding the conceptual rules in respect to the logic of number/letter patterns).

Four exercises were devoted to attention training than other cognitive abilities since it is a commonly observed deficit after severe malaria. Captain's Log was programmed to run for 45 minutes with the first training session starting at the simplest level and the difficulty increased based on the child's performance. Children performed two sessions once a week for eight weeks.

### Assessments

#### Kaufman Assessment Battery for Children second edition (KABC-II)

The KABC-II is a comprehensive assessment of cognitive ability containing a number of scales that have been adapted, piloted and validated among African children in Kenya [4], Senegal [5] and Uganda [8]. It retains its construct validity when used in Ugandan children to access Working Memory (taking in and holding information and then using it within a few seconds), Visual Spatial ability (perceiving, storing, manipulating and thinking with visual patterns), Learning (storing and efficiently retrieving newly learned or previously learned information) and Planning (solving novel problems by using reasoning abilities like induction and deduction) [33].

#### Test of Variables of Attention (TOVA)

The TOVA is a computer administered visual continuous performance test used in the diagnosis and monitoring of children and adults with attention deficit disorders [34]. The TOVA has been used in previous malaria studies in Senegal and Uganda [5,8,9]. It is a sensitive measure of cerebral insult from malaria as indicated by the persisting attention deficits at 24 months in Ugandan children with cerebral malaria [9]. The outcome measure was the d' prime score, which is the child's accuracy in discriminating between the target and non-target stimuli.

#### Child Behaviour Checklist (CBCL)

The CBCL is a paper-pencil child behavioural rating scale consisting of 120 items to which a parent/guardian responds [35]. It has been validated in 30 societies, has proven useful in multicultural assessment of children [36] and has fair reliability in Ugandan children [12]. Studies examining the structure of psychopathology have identified internalising and externalising symptoms as the two broad factors underlying psychopathology [37,38]. The externalising factor is comprised of substance dependence, attention-deficit hyperactivity disorder, oppositional defiant disorder, and conduct disorder/antisocial personality disorder. The internalising factor has 'distress/misery' comprising generalised anxiety disorder, overanxious disorder and depressive disorders and 'fear', which includes simple and social phobias, separation anxiety disorder and panic disorder [37]. These two broad categories of internalising and externalising psychopathologic behaviour are measured by the CBCL and were the outcomes for behaviour in this study.

#### Wide Range Achievement Test-third edition (WRAT-3)

The WRAT-3 is a measure of the codes needed to learn the basic skills of reading, spelling and arithmetic [39,40]. It has been used earlier for research in Ugandan children with HIV [41].

#### Middle Childhood Home Observations for the Measurement of the Environment (MC-HOME)

The MC-HOME [42] identifies parental behaviours that are important to children's cognitive development and academic skills. The MC-HOME as adapted to the Ugandan setting by Boivin and colleagues [8] was used in this study. It has 58 items measuring the amount of stimulation and learning opportunities available to the child in the home, summed into a single score. The MC-HOME measures the quality of the home environment that is predictive of working memory performance in Ugandan children [43].

Since attention was shown as the persisting deficit at 24 months in Ugandan children [9], attention capacity measured by the TOVA was the primary outcome of this study. Attention deficits in severe malaria may affect other cognitive skills that are dependent on it which involve vigilance and perceptual acuity [5]. These other cognitive skills were the secondary outcomes for cognition; working memory, visual spatial ability, reasoning and learning. For academic skills, reading, spelling and arithmetic were the outcomes and for behaviour, they were internalising and externalising problems.

### Data analysis

Data was analysed using SPSS 17.0. Log transformation was done where appropriate for variables that were not normally distributed prior to statistical analysis. To assess the effect of the intervention while controlling for other covariates, analysis of covariance (ANCOVA) was run on the post-intervention score with covariates age, weight for age z scores, quality of the home environment, sex, time between admission and post-intervention assessment and baseline pre-intervention score on the same outcome variable. Data are presented as estimated means (with standard errors) between the intervention and control groups.

## Results

Children in the treatment and non-treatment groups had similar demographic characteristics as shown in table 1. Importantly, there was a similar distribution of children with cerebral malaria, malaria with seizures and malaria with impaired consciousness between the treatment and control groups. The mean period between the date of admission and the post intervention testing was approximately seven months (range 5.43 to 10.47 months). At baseline, both groups had similar cognitive, academic and behavioural scores (Table 2). In addition, there were no differences in the outcomes scores between the three different malaria groups (data not shown).

**Table 1:** Participants' demographic characteristics

Domain	Control group N=33	CCRT group N=28	*P*
Gender, male n (%)	19 (52.78)	17 (57.78)	0.80
Age (years)	7.39 (1.84)	7.08 (1.52)	0.49
School grade	2.64 (1.78)	2.14 (1.21)	0.22
Weight (kgs)	21.51 (4.99)	21.29 (5.60)	0.87
Height (cm)	119.88 (15.58)	118.50 (13.71)	0.76
Weight for age z score	-0.98 (1.12)	-0.92 (1.22)	0.86
Home environment score	24.14 (6.33)	22.65 (6.22)	0.36
Interval between admission and post-testing (days)	199.30 (19.50)	210.96 (38.04)	0.13
Had cerebral malaria n (%)	3 (9.1)	5 (17.9)	0.45
Had malaria with seizures n (%)	19 (57.6)	15 (53.6)	0.75
Had malaria with impaired consciousness n (%)	11 (33.3)	8 (28.6)	0.69

cm = centimetres, kgs = kilograms, N = Number. All values are Mean (SD) unless otherwise stated.

Comparisons made using t-tests and chi-square where appropriate

**Table 2:** Baseline scores^1 ^between the two groups

Domain	Control group N=33	CCRT group N=28	*P*
Attention^a^	1.90 (0.14)	1.79 (0.15)	0.57
Working memory	25. 44 (1.02)	26.02 (1.11)	0.70
Visual spatial ability	30.67 (1.88)	28.28 (2.04)	0.40
Reasoning^a^	1.89 (0.09)	1.70 (0.10)	0.17
Learning^a^	4.36 (0.07)	4.26 (0.07)	0.31
Arithmetic	12.13 (0.86)	12.22 (0.97)	0.94
Reading	12.23 (1.10)	13.50 (1.22)	0.45
Spelling	11.38 (1.15)	13.58 (1.27)	0.21
Internalising Problems^a^	2.43 (0.10)	2.54 (0.11)	0.45
Externalising Problems^a^	2.70 (0.11)	2.79 (1.12)	0.61

^1^Mean scores for control and treatment groups, adjusted for age, sex, quality of the home environment, weight for age z score by ANCOVA.

^a^Log transformed scores

All values are Mean (Standard error)

Intervention effects were observed in learning mean score (SE) [93.89 (4.00) vs 106.38 (4.32), *P *= 0.04]. For working memory however, the effect showed improvement in the control group and not the treatment group [27.42 (0.66) vs 25.34 (0.73), *P *= 0.04. No intervention effects were observed in attention, the primary outcome or in any of the other cognitive outcomes or in academic skills and behavioural measures as shown in Table 3.

**Table 3:** Mean scores post-intervention between intervention and control groups

Domain	Control group N=33	CCRT group N=28	*P*
Attention^a^	2.00 (0.15)	1.89 (0.17)	0.64
Working memory	27.42 (0.66)	25.34 (0.73)	**0.04**
Visual spatial ability	36.48 (1.41)	36.13 (1.55)	0.87
Reasoning^a^	2.10 (0.10)	2.06 (0.10)	0.76
Learning	93.89 (4.00)	106.38 (4.32)	**0.04**
Arithmetic	13.97 (0.55)	12.55 (0.64)	1.00
Reading^a^	2.18 (0.08)	2.45 (0.09)	0.58
Spelling^a^	2.22 (0.12)	2.23 (0.14)	0.96
Internalising Problems^a^	2.53 (0.10)	2.50 (0.11)	0.84
Externalising Problems	15.99 (1.28)	17.23 (1.40)	0.52

ANCOVA, adjusted for age, weight for age z score, quality of the home environment, sex, pre-intervention score and time between admission and post-intervention assessment.

^a^Log transformed scores

All values are Mean (Standard error)

There were interaction effects between treatment group and sex for Arithmetic only with the boys in the intervention group having a better score than those in the control arm (mean difference in adjusted scores between intervention and control group (standard error) 1.13 (0.81): *P *= 0.17). For the girls, those in the intervention group had poorer scores than those in the control arm (-3.63 (1.94): *P *= 0.08).

## Discussion

The aim of this study was to investigate the effect of CCRT on cognition, academic skills and behaviour in children after an episode of severe malaria. Analyses controlling for possible confounders found an intervention benefit in learning.

Our present findings of some benefit on cognition from CCRT are similar to recent findings from Uganda done among children with cerebral malaria and HIV showing improvement in some cognitive domains after the intervention [19,29]. Both these studies showed an improvement in learning as we did. The current study however showed a positive benefit in only one outcome out of the ten tested. In addition, the intervention group had poorer working memory scores after the intervention. In some of the recent successful CCRT studies with children, more training sessions were done than the 16 sessions in our study, which may explain why minimal effect was seen as the intervention was not intense [15,16,20,44].

Improvement in some outcomes may however not be evident if assessed within a month after the intervention as we did. Holmes at al did not notice any benefit in academic skills a week after cognitive training in children but noticed a benefit at six months instead [44]. They concluded that any cognitive support to learning caused by training would be expected to take some time to work before any benefits are observed.

In other studies, CCRT has been shown to improve cognitive functioning in children with different forms of brain injury, including cerebral malaria, HIV, attention deficit hyperactivity disorder and brain tumours [16,17,19,20,29]. Some of these studies showed benefits at three and six months follow up [16,20]. We can't readily state whether our intervention's benefits will be sustained in these children with malaria since we have not done follow-up evaluations. Improvements in cognition after training have been associated with physiological changes in the brain that are linked with cognitive development [21-24]. In addition, cognitive training has improved other skills that are not trained like academic scores and everyday behaviour [16,17,29,44]. We did not see differences in academic scores or behaviour scores in our study, possibly because the selected cognitive exercises in our program are not effective for these outcomes.

It is not yet clear whether CCRT at three months or at four years for severe malaria survivors will produce better results. In our two studies with severe malaria survivors, CCRT at four years had better results than the current study with an earlier intervention. However, since all the post-intervention assessments were immediate, we cannot draw firm conclusions from these studies about optimal timing of interventions for sustained benefit.

A major limitation of this study was the design of the intervention program. Whereas our cognitive intervention studies have used the same cognitive regimen training several abilities, other studies have instead trained only working memory but still shown benefits in other cognitive skills [16,44]. Training a specific cognitive ability might produce better results than what we have observed in our studies because more time is given to training a specific individual ability. In addition, accomplishing 16 training sessions was intensive in our study area, but this number of sessions is fewer than what some other effective studies have used [15,16,20,44]. It is therefore important to establish what skills to train and how many sessions are adequate for a detectable benefit to be observed.

Other limitations of this study include; no follow up several months later, no active control group, a small sample size and lack of blinding of assessors to the children's treatment group. A larger trial with a careful study design is currently being set up to address some of the above limitations.

## Conclusions

We conclude that our cognitive intervention targeting different cognitive abilities may improve some cognitive abilities but not academic skills and behaviour three months after severe malaria. Future studies need to assess whether the effects of computerised cognitive rehabilitation training in children with severe malaria are sustained over time.

## Competing interests

The authors declare that they have no competing interests.

## Authors' contributions

PB conceived the study, participated in the data collection, analysis, interpretation and writing of the manuscript. PA, MJB, CCJ, AE and SM participated in the interpretation and writing of the manuscript. CP participated in the, analysis, interpretation and writing of the manuscript. All authors read and gave approval of the final version to be published.

## Pre-publication history

The pre-publication history for this paper can be accessed here:

http://www.biomedcentral.com/1471-2377/11/96/prepub
